# Association of the remnant cholesterol inflammation index with long-term outcomes among patients with acute ischemic stroke treated with intravenous thrombolysis

**DOI:** 10.3389/fendo.2026.1984138

**Published:** 2026-11-05

**Authors:** Yuan Cheng, Qingqing Wang, Jinhua Liao

**Affiliations:** 1 Fuyang Medical College, Fuyang Normal University, Fuyang, China; 2 Department of Neurology, The Affiliated Fuyang People’s Hospital of Anhui Medical University, Fuyang, China; 3 Department of Medical Oncology, Fuyang Cancer Hospital, Affiliated Fuyang Cancer Hospital of Fuyang Normal University, Fuyang, China

**Keywords:** all-cause mortality, ischemic stroke, recurrent ischemic stroke, remnant cholesterol inflammation index, thrombolysis

## Abstract

**Background:**

The Remnant Cholesterol Inflammation Index (RCII) is a novel assessment tool that reflects lipid metabolism disorders and systemic inflammation, and may be associated with adverse vascular outcomes. This study aimed to investigate the relationship between RCII and long-term outcomes in patients with acute ischemic stroke (AIS) after intravenous thrombolysis (IVT).

**Methods:**

We conducted a cohort study of 748 patients with AIS who received IVT between April 2019 and March 2025 and survived the index hospitalization. All patients were divided into four groups based on log-transformed RCII (lnRCII), and the endpoint events studied were recurrent ischemic stroke and all-cause mortality. Patients were followed from hospital discharge until March 31, 2026. We used survival and competing-risk analyses, Cox proportional hazards models, and restricted cubic splines to evaluate the associations between lnRCII and long-term outcomes.

**Results:**

The median age was 68 years, the interquartile range was 59–76 years, 63.6% were male. During the follow-up period, recurrent ischemic stroke was confirmed in 101 (13.5%) patients, and all-cause mortality was confirmed in 120 (16.0%) patients. Multivariable Cox analysis showed that higher lnRCII levels were significantly associated with increased risks of recurrent ischemic stroke (HR = 1.373, 95% CI: 1.174-1.605, P < 0.001) and all-cause mortality (HR = 1.275, 95% CI: 1.107-1.469, P < 0.001). Restricted cubic spline analyses demonstrated no significant evidence of nonlinearity in the associations between lnRCII and either outcome (P for nonlinearity > 0.05). Sensitivity analyses yielded generally consistent findings. Exploratory subgroup analyses identified significant interactions with age and hypertension for all-cause mortality.

**Conclusions:**

In patients with AIS treated with IVT, elevated RCII was independently associated with an increased risk of long-term recurrent ischemic stroke and all-cause mortality. RCII may prove to be a composite marker associated with long-term outcomes.

## Introduction

In recent years, the incidence of acute ischemic stroke (AIS) has exhibited a complex pattern characterized by a steadily increasing global disease burden, significant regional variations, and a trend toward younger onset (1–4). Although intravenous thrombolysis (IVT) has been widely used as the standard treatment for AIS and has demonstrated significant clinical efficacy, the time-sensitive nature of IVT and its low recanalization rates mean that some patients still experience severe neurological deficits (5, 6). Therefore, determining how to accurately assess the individual risk of recurrent ischemic stroke and mortality in patients with AIS receiving IVT has become a key area of research for improving clinical outcomes, optimizing secondary prevention strategies, and reducing adverse risks to patients.

Remnant cholesterol (RC) refers to the cholesterol component found in triglyceride-rich lipoproteins (7, 8). Studies have demonstrated that for each one-standard-deviation increase in RC levels, the risk of stroke increases by approximately 6% (9). At the same time, C-reactive protein (CRP), a classic biomarker of systemic inflammation, has been shown to be significantly associated with poor clinical outcomes, increased infarct volume, and stroke severity when elevated (10, 11). The Remnant Cholesterol Inflammation Index (RCII), a recently proposed composite biomarker integrating remnant cholesterol and inflammatory markers, may reflect the combined burden of dyslipidemia and systemic inflammation associated with atherosclerosis (8, 12, 13). Previous studies have investigated the association of RCII with stroke risk and clinical outcomes (12, 13). A study based on the CHARLS database also showed that for every one-standard-deviation increase in RCII, the risk of stroke rises by 10.6% (12). However, evidence regarding the associations of RCII with long-term recurrent ischemic stroke and all-cause mortality among patients with AIS treated with IVT remains limited. Therefore, we conducted a cohort study to investigate the associations of RCII with long-term recurrent ischemic stroke and all-cause mortality in patients with AIS treated with IVT.

## Materials and methods

### Research design and study population

We conducted this study at The Affiliated Fuyang People’s Hospital of Anhui Medical University, which consecutively enrolled patients with AIS who were treated with IVT between April 2019 and March 2025. All patients were diagnosed with AIS based on clinical presentation and neuroimaging findings and met the criteria for IVT treatment. Before IVT, neuroimaging assessment was performed using CT or MRI, while MRI was performed either before or after IVT to support the diagnosis of AIS. Alteplase was administered at 0.9 mg/kg (maximum 90 mg), with 10% as a bolus and the remainder over 60 minutes. Exclusion criteria included: patients with malignancies, or autoimmune diseases; those with severe hepatic or renal impairment; those who died during the hospitalization; those with missing key clinical or laboratory data; those with unavailable follow-up information; and bridging IVT followed by endovascular thrombectomy. Of the 975 patients initially screened, 227 were excluded: 32 for severe hepatic or renal dysfunction, 27 for malignancies or autoimmune diseases, 30 for in-hospital death, 70 for missing key clinical or laboratory data, 22 for unavailable follow-up information, and 46 for bridging therapy, leaving 748 patients in the final analysis (**Figure 1****).** The present study focused on long-term post-discharge outcomes among patients who received IVT and survived hospitalization.

**Figure 1:**
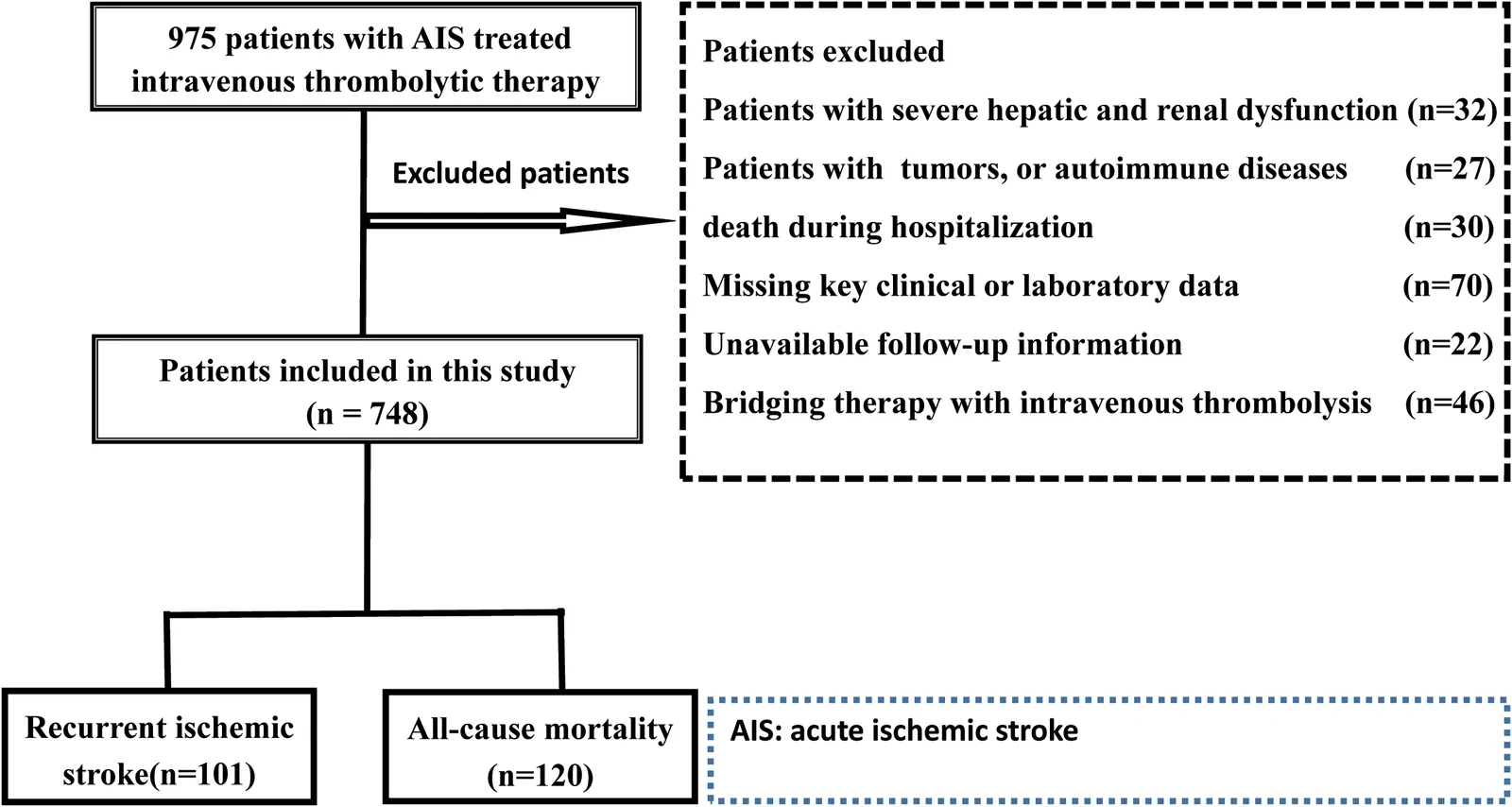
Study flowchart of the present study. Recurrent ischemic stroke and all-cause mortality were assessed as separate endpoints in the same cohort, and the outcome groups were not mutually exclusive.

### Data collection

Clinical and laboratory data were retrospectively abstracted from routine medical records. Baseline clinical data included demographic characteristics and cerebrovascular risk factors, and onset-to-needle time (ONT). Upon admission, all patients underwent assessment of the severity of neurological deficits using the National Institutes of Health Stroke Scale (NIHSS). The modified Rankin Scale (mRS) score was assessed at hospital discharge. Subtypes of stroke were categorized by large-artery disease (LAD), Small-artery disease (SAD), Cardiac embolism (CE), and others (other and undetermined etiology) (14). Fasting venous blood samples were collected on the morning following admission, after completion of IVT. Laboratory measurements included lipid profile [total cholesterol (TC), triglycerides (TG), low-density lipoprotein cholesterol (LDL-C), and high-density lipoprotein cholesterol (HDL-C)], CRP, and fasting blood glucose (FBG). These laboratory parameters were measured using an ADVIA 2400 automated chemistry analyzer (Siemens, Germany). LDL-C was directly measured rather than calculated. TC, TG, LDL-C, HDL-C, and FBG were reported in mmol/L, whereas CRP was reported in mg/L.

### Definition of the remnant cholesterol inflammation index

RC was calculated based on lipid profile measurements using the formula: RC = TC – (LDL-C + HDL-C). Because TC, LDL-C, and HDL-C were measured in mmol/L, the calculated RC was subsequently converted to mg/dL using a conversion factor of 38.67 [RC (mg/dL) = RC (mmol/L) × 38.67]. RCII was then calculated as RCII = RC (mg/dL) × CRP (mg/L)/10. All calculated RCII values were greater than zero; therefore, no offset or other correction was required before logarithmic transformation. To mitigate the impact of skewed distributions on statistical analysis, RCII is log-transformed (lnRCII) when analyzed as a continuous variable (8, 15). As a worked example, for a de-identified patient with TC of 3.81 mmol/L, LDL-C of 2.07 mmol/L, HDL-C of 0.94 mmol/L, and CRP of 3.87 mg/L, RC was calculated as 0.80 mmol/L and subsequently converted to 30.94 mg/dL. The corresponding RCII was 11.97, yielding an lnRCII value of 2.483.

### Outcome measures and follow-up

The primary endpoints of this study were recurrent ischemic stroke and all-cause mortality. Follow-up for long-term recurrent ischemic stroke and all-cause mortality commenced at hospital discharge, which was defined as the time origin for the time-to-event analyses. After enrolling patients with AIS who received IVT, the study conducted follow-up assessments every 6 months via telephone interviews or outpatient visits. The follow-up cutoff date for this analysis was March 31, 2026. Recurrent ischemic stroke was defined as new or worsening neurological deficits with a new ischemic lesion confirmed by neuroimaging. Hemorrhagic stroke, transient ischemic attack, and events occurring during the index hospitalization were not considered recurrent events. Neuroimaging confirmation was used to distinguish recurrent ischemic stroke from progression of the index stroke, hemorrhagic transformation, or other post-stroke complications. All-cause mortality referred to death from any cause, confirmed through outpatient follow-up records, telephone follow-up, or death registry data. All outcome assessments were reviewed by medical professionals.

### Statistical analysis

Descriptive statistics summarized baseline data as frequencies, means ± SD/medians, or median (M) and interquartile range (IQR). All participants were grouped by quartiles based on their lnRCII distribution. For recurrent ischemic stroke, cumulative incidence functions were estimated with death before recurrent ischemic stroke treated as a competing event, and differences across lnRCII quartiles were assessed using Gray’s test. Cox proportional hazards models were used for the primary analysis, and Fine–Gray models were additionally performed to account for the competing risk of death. Event-free participants were censored at the last follow-up or study end; for recurrent ischemic stroke analyses, death before recurrent ischemic stroke was treated as a censoring event in Cox models. For all-cause mortality, Kaplan–Meier and Cox proportional hazards analyses were used. Absolute risks of recurrent ischemic stroke at 12 and 24 months were estimated using the cumulative incidence function, accounting for death as a competing event. Corresponding all-cause mortality risks were estimated as 1 minus the Kaplan–Meier survival probability, with 95% confidence intervals calculated for both outcomes. Median follow-up duration was estimated using the reverse Kaplan–Meier method. The proportional hazards assumption was assessed using Schoenfeld residuals and was not violated (all P > 0.05). Cox proportional hazards models were used to evaluate the associations of lnRCII with recurrent ischemic stroke and all-cause mortality, with hazard ratios (HRs) and 95% confidence intervals (CIs) reported. Covariates for the multivariable models were selected based on their clinical relevance and potential confounding relationships with RCII and long-term outcomes. Model I was unadjusted; Model II was adjusted for age and gender; and Model III was adjusted for age, gender, baseline NIHSS score, stroke etiology, hypertension, diabetes mellitus, ischemic heart disease, atrial fibrillation, and history of stroke. Additional fully adjusted Cox regression models using the same covariates as Model III were performed to compare lnRCII with its individual components. lnRCII, RC, and lnCRP were evaluated separately, and RC and lnCRP were also entered simultaneously; CRP was natural-log transformed because of its right-skewed distribution. We also performed subgroup analyses by potential confounders. These analyses were considered exploratory, and no adjustment for multiple comparisons was performed. Restricted cubic spline (RCS) analyses with three knots at the 10th, 50th, and 90th percentiles were performed to assess the dose-response and potential nonlinear associations, with lnRCII = 2.312 as the reference value. Sensitivity analyses were performed separately after excluding patients with clinically documented severe infection during hospitalization (n = 20) or extreme lnRCII values (n = 4), defined using the 1.5 × interquartile range rule (lnRCII > 5.788). Severe infection was identified based on documented clinical diagnoses supported by relevant clinical findings, laboratory results, and/or imaging evidence, with severity determined according to clinical assessments recorded during hospitalization rather than isolated elevations in inflammatory markers. The timing of infection was assessed relative to baseline blood sampling performed on the morning following admission after IVT. All 20 cases of severe infection developed after baseline blood sampling. All sensitivity analyses were adjusted using the same covariates as Model III. All statistical analyses were performed using R software (version 4.1.2). Two-sided P-value of <0.05 was considered statistically significant.

## Results

### Clinical data

This study included a total of 748 patients with AIS who underwent IVT. Among them, 476 were male (63.6%), with a median age of 68 years (IQR: 59–76). The median follow-up duration was 36.3 months (95% CI, 33.5–38.5 months). The lnRCII values ranged from −0.755 to 6.168. Patients were categorized into quartiles according to lnRCII levels [quartile (Q) 1, ≤1.353; Q2, >1.353 to ≤2.308; Q3, >2.308 to ≤3.127; and Q4, >3.127], and the baseline characteristics of each quartile are summarized in **Table 1**. Compared with the lowest quartile, patients in the highest quartile had higher prevalences of vascular risk factors such as diabetes and dyslipidemia, significantly higher NIHSS scores at admission, as well as significantly elevated levels of inflammatory markers (CRP) and remnant cholesterol (all P < 0.05). Furthermore, patients in the higher lnRCII quartiles tended to have higher fasting blood glucose levels.

**Table 1:** Baseline characteristics stratified across quartiles of lnRCII.

Variables	Total (n = 748)	Q1 (n=186)	Q2 (n=188)	Q3 (n=187)	Q4 (n=187)	P value
Age (years)	68 (59-76)	68 (57-75)	67 (58-74)	68 (60-76)	70 (61-78)	0.099
Male, *n* (%)	476 (63.6)	113 (60.8)	121 (64.4)	124 (66.3)	118 (63.1)	0.726
Risk factors, *n* (%)						
Hypertension	562 (75.1)	128 (68.8)	147 (78.2)	141 (75.4)	146 (78.1)	0.123
Diabetes	183 (24.5)	30 (16.1)	46 (24.5)	50 (26.7)	57 (30.5)	0.011
Dyslipidemia	266 (35.6)	42 (22.6)	67 (35.6)	70 (37.4)	87 (46.5)	< 0.001
Ischemic heart disease	123 (16.4)	23 (12.4)	29 (15.4)	32 (17.1)	39 (20.9)	0.164
Atrial fibrillation	105 (14.0)	23 (12.4)	27 (14.4)	32 (17.1)	23 (12.3)	0.497
History of stroke	166 (22.2)	36 (19.4)	38 (20.2)	46 (24.6)	46 (24.6)	0.467
Smoker	270 (36.1)	62 (33.3)	64 (34.0)	70 (37.4)	74 (39.6)	0.555
Alcohol user	265 (35.4)	59 (31.7)	64 (34.0)	75 (40.1)	67 (35.8)	0.380
Laboratory findings						
TC (mmol/L)	4.54±1.07	4.19±1.04	4.50±0.96	4.66±0.98	4.82±1.19	< 0.001
TG (mmol/L)	1.17 (0.86-1.62)	0.99 (0.76-1.34)	1.16 (0.87-1.62)	1.22 (0.95-1.62)	1.39 (0.96-1.90)	< 0.001
HDL (mmol/L)	1.09 (0.93-1.29)	1.15 (0.98-1.39)	1.11 (0.93-1.28)	1.08 (0.94-1.25)	1.06 (0.89-1.19)	0.002
LDL (mmol/L)	2.56±0.84	2.32±0.83	2.53±0.74	2.65±0.78	2.72±0.93	< 0.001
FBG (mmol/L)	5.32 (4.75-6.13)	5.04 (4.62-5.66)	5.34 (4.77-6.11)	5.32 (4.81-6.29)	5.56 (4.82-6.59)	< 0.001
CRP (mg/L)	3.19 (1.31-7.44)	0.88 (0.59-1.16)	2.08 (1.49-2.93)	5.22 (3.73-7.45)	10.08 (7.45-20.75)	< 0.001
RC (mg/dL)	30.16 (23.39-38.47)	25.90 (19.72-30.71)	30.16 (23.97-39.05)	30.55 (24.17-38.67)	35.18 (27.65-43.69)	< 0.001
lnRCII	2.29±1.26	0.69±0.49	1.84±0.28	2.75±0.25	3.86±0.75	< 0.001
Onset-to-needle time, min	149 (111-200)	159 (120-210)	147 (114-200)	153 (116-198)	141(107-197)	0.242
NIHSS	5 (3-8)	5 (3-6)	4 (3-8)	5 (3-9)	5 (3-9)	0.036
mRS	1 (0-2)	1 (0-2)	1 (0-2)	1 (1-3)	1 (0-3)	< 0.001
**Stroke etiology, n (%)**						0.762
LAD	306 (40.9)	73 (39.2)	75 (39.9)	81 (43.3)	77 (41.2)	
CE	90 (12.0)	21 (11.3)	18 (9.6)	29 (15.5)	22 (11.8)	
SAD	221 (29.5)	57 (30.6)	58 (30.9)	49 (26.2)	57 (30.5)	
Other and undetermined etiologies	131 (17.5)	35 (18.8)	37 (19.7)	28 (15.0)	31 (16.6)	

TC, total cholesterol; TG, triglyceride; HDL, high-density lipoprotein cholesterol; LDL, low-density lipoprotein cholesterol; FBG, fasting blood glucose; CRP, C-reactive protein; RC, remnant cholesterol; RCII, remnant cholesterol inflammation index; NIHSS, National Institutes of Health Stroke Scale; mRS, modified Rankin Scale; LAD, large artery disease; CE, cardiac embolism; SAD, small artery disease. P values for comparisons across lnRCII quartiles were calculated using one-way analysis of variance or the Kruskal–Wallis test for continuous variables, as appropriate, and the chi-square test or Fisher’s exact test for categorical variables, as appropriate.

### Analysis of the risk of recurrent ischemic stroke following IVT in patients with AIS

Recurrent ischemic stroke was observed in 101 patients (13.5%) during follow-up. Cumulative incidence analysis, with death before recurrent ischemic stroke treated as a competing event, showed significant differences in recurrent ischemic stroke across lnRCII quartiles (Gray’s test P < 0.001) (**Figure 2A**). At 12 and 24 months, the cumulative incidence of recurrent ischemic stroke, accounting for death as a competing event, was 5.76% (95% CI, 4.15%–7.49%) and 9.71% (95% CI, 7.57%–11.95%), respectively. Results from multivariable Cox regression analysis indicated that elevated lnRCII levels were significantly associated with the risk of recurrent ischemic stroke (HR = 1.373, 95% CI: 1.174-1.605, P < 0.001). Analysis by quartile showed that, compared with Q1, patients in the Q4 had a significantly increased risk of recurrent ischemic stroke (HR = 3.158, 95% CI: 1.593-6.260, P < 0.001) (**Table 2**). These findings remained consistent in competing-risk analyses accounting for death before recurrent ischemic stroke (adjusted sHR, 1.341; 95% CI, 1.161–1.550; P < 0.001) (**Supplementary Table S1**).

**Table 2:** Multivariate Cox proportional hazards models for the risk of recurrent ischemic stroke.

Variables	Model I		Model II		Model III	
	HR (95% CI)	P value	HR (95% CI)	P value	HR (95% CI)	P value
lnRCII	1.499 (1.289-1.742)	< 0.001	1.438 (1.239-1.669)	< 0.001	1.373 (1.174-1.605)	< 0.001
lnRCII, Quartiles						
Q1	Ref		Ref		Ref	
Q2	2.099 (1.012-4.355)	0.046	2.139 (1.031-4.438)	0.041	2.033 (0.974-4.240)	0.059
Q3	3.044 (1.530-6.056)	0.002	3.137 (1.575-6.247)	0.001	2.646 (1.318-5.314)	0.006
Q4	3.989 (2.038-7.809)	< 0.001	3.767 (1.923-7.380)	< 0.001	3.158 (1.593-6.260)	< 0.001

All models included 748 patients, with 101 recurrent ischemic stroke events. Model I: Unadjusted model; Model II: adjusted for gender, age; Model III: adjusted for age, gender, baseline NIHSS score, stroke etiology, hypertension, diabetes mellitus, ischemic heart disease, atrial fibrillation, and history of stroke. HRs for continuous lnRCII are expressed per one-unit increase in lnRCII. CI, confidence interval; RCII remnant cholesterol inflammation index; HR, hazard ratio.

**Figure 2:**
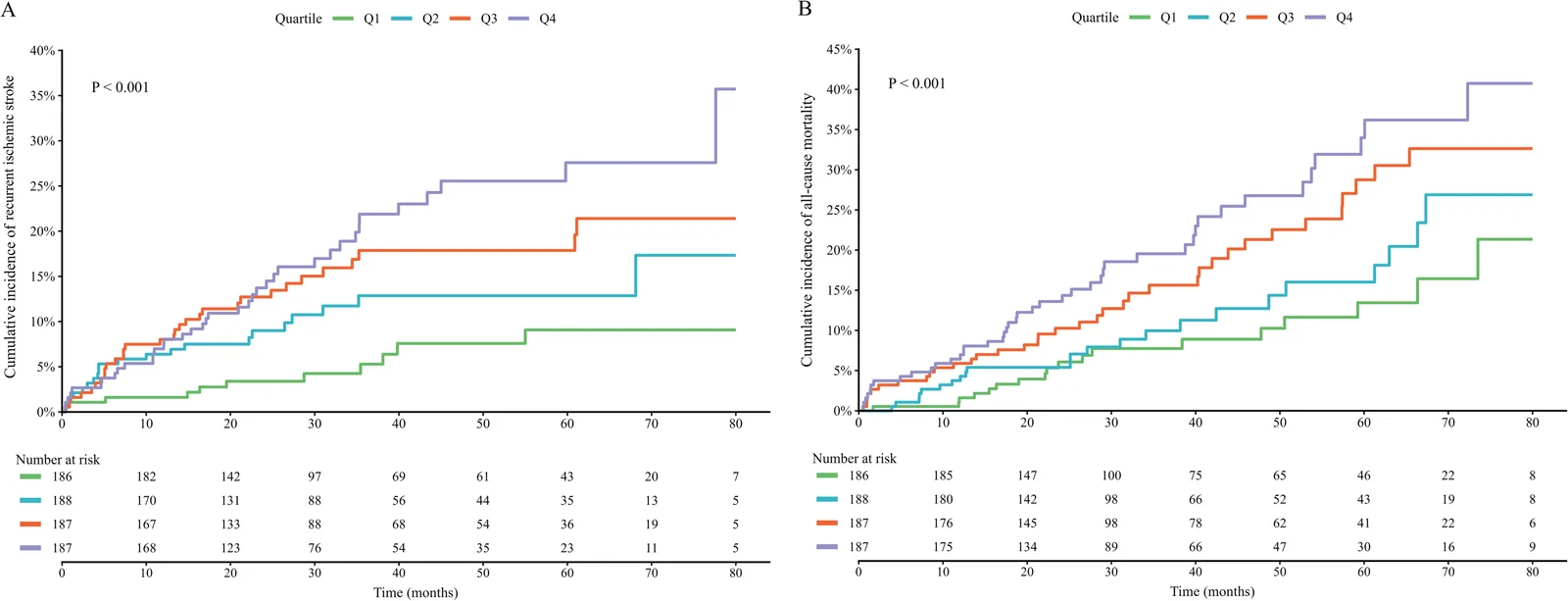
Cumulative incidence curves for recurrent ischemic stroke **(A)**, with death before recurrent ischemic stroke treated as a competing event, and cumulative probability of all-cause mortality estimated as 1 minus the Kaplan–Meier survival probability (1 − KM) **(B)** according to lnRCII quartiles. Numbers at risk indicate participants remaining at risk at each time point. Estimates at later follow-up times should be interpreted cautiously because few patients remained at risk.

### Analysis of all-cause mortality risk following IVT in patients with AIS

A total of 120 deaths (16.0%) occurred during follow-up. Kaplan–Meier survival analysis showed significant differences in the incidence of all-cause mortality across lnRCII quartiles (log-rank P < 0.001) (**Figure 2B**). The cumulative probabilities of all-cause mortality at 12 and 24 months were 4.43% (95% CI, 3.17%–6.17%) and 8.87% (95% CI, 6.97%–11.25%), respectively. Results from the Cox proportional hazards model analysis indicated that elevated lnRCII levels were significantly linked to an increased risk of all-cause mortality (HR = 1.275, 95% CI: 1.107-1.469, P < 0.001). Analysis by quartile further confirmed that, compared with Q1, patients in Q4 had a significantly increased risk of all-cause mortality (HR = 1.948, 95% CI: 1.092-3.473, P = 0.024) (**Table 3**).

**Table 3:** Multivariate Cox proportional hazards models for the risk of all-cause mortality after acute ischemic stroke.

Variables	Model I		Model II		Model III	
	HR (95% CI)	P value	HR (95% CI)	P value	HR (95% CI)	P value
lnRCII	1.505 (1.306-1.733)	< 0.001	1.372 (1.202-1.556)	< 0.001	1.275 (1.107-1.469)	< 0.001
lnRCII, Quartiles						
Q1	Ref		Ref		Ref	
Q2	1.377 (0.743-2.553)	0.309	1.535 (0.827-2.849)	0.174	1.445 (0.766-2.724)	0.255
Q3	2.063 (1.172-3.634)	0.012	2.477 (1.402-4.374)	0.002	1.942 (1.085-3.477)	0.026
Q4	2.737 (1.578-4.747)	< 0.001	2.550 (1.467-4.430)	0.001	1.948 (1.092-3.473)	0.024

All models included 748 patients, with 120 all-cause mortality events.

Model I: Unadjusted model; Model II: adjusted for gender, age; Model III: adjusted for age, gender, baseline NIHSS score, stroke etiology, hypertension, diabetes mellitus, ischemic heart disease, atrial fibrillation, and history of stroke. HRs for continuous lnRCII are expressed per one-unit increase in lnRCII. CI, confidence interval; RCII remnant cholesterol inflammation index; HR, hazard ratio.

### Associations of individual components with long-term outcomes

In additional analyses using the same fully adjusted covariate set, lnRCII, RC, and lnCRP were each significantly associated with recurrent ischemic stroke. When RC and lnCRP were simultaneously included in the model, both remained significantly associated with recurrent ischemic stroke (**Supplementary Table S2**). For all-cause mortality, lnRCII and lnCRP were significantly associated with mortality, whereas RC was not. When RC and lnCRP were simultaneously included, lnCRP remained significantly associated with mortality, whereas RC did not (**Supplementary Table S3**).

### Stratified analyses and restricted cubic spline

Subgroup analyses (**Figure 3**) showed that the associations of lnRCII with recurrent ischemic stroke and all-cause mortality were generally consistent across most subgroups. For recurrent ischemic stroke, no statistically significant interactions were observed across the examined subgroups (all P for interaction ≥ 0.05). For all-cause mortality, significant interactions were observed for age (P for interaction < 0.001) and hypertension (P for interaction = 0.045), whereas no significant interactions were observed for the other subgroup variables.

**Figure 3:**
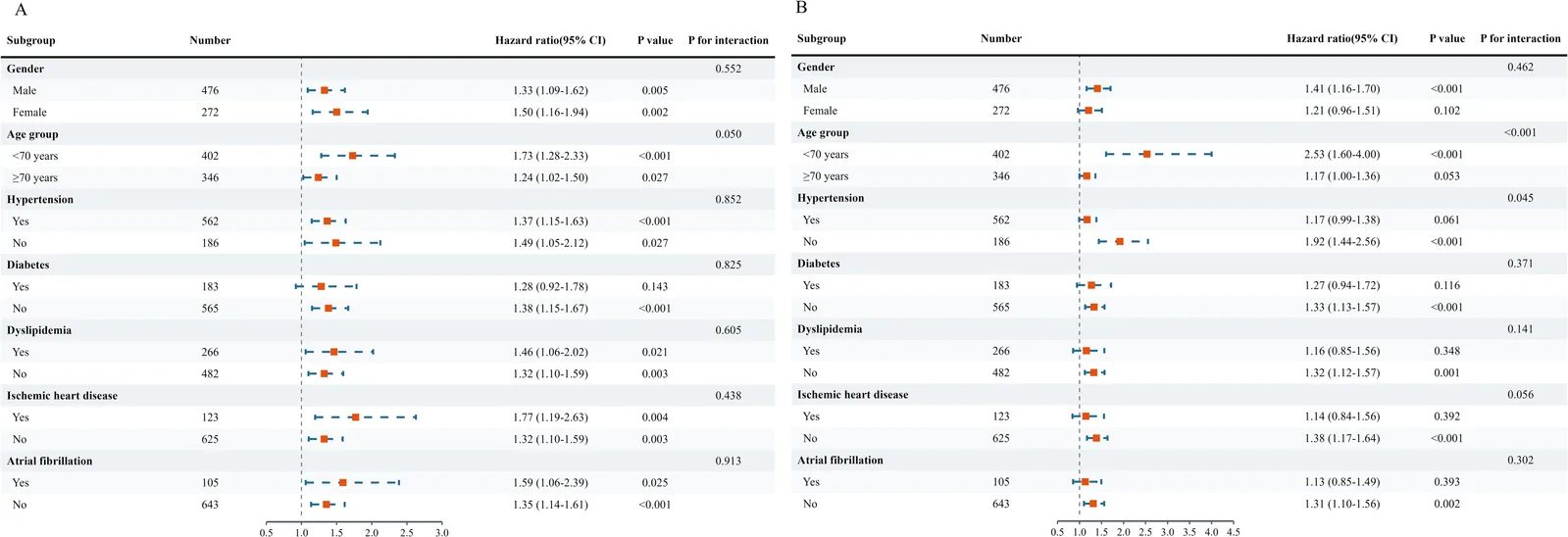
Subgroup analyses of the associations between lnRCII and the risks of recurrent ischemic stroke **(A)** and all-cause mortality **(B)** among patients with AIS treated with intravenous thrombolysis. HRs and 95% CIs were estimated using Cox proportional hazards regression models adjusted for age, gender, baseline NIHSS score, stroke etiology, hypertension, diabetes mellitus, ischemic heart disease, atrial fibrillation, and history of stroke, except for the corresponding stratification variable. HRs are expressed per one-unit increase in lnRCII.

To provide a clearer and more intuitive visualization of the relationship between lnRCII and the risk of recurrent ischemic stroke and all-cause mortality, we used Cox regression analysis to construct a RCS curve. In the RCS analysis fully adjusted for confounding factors, the curve showed that elevated lnRCII was associated with an increasing trend in the long-term risk of recurrent ischemic stroke, with no significant evidence of nonlinearity (P for nonlinearity = 0.546) (**Figure 4A**). Furthermore, higher lnRCII values in patients with AIS were associated with an increased risk of all-cause mortality, with no significant evidence of nonlinearity (P for nonlinearity = 0.462) (**Figure 4B**).

**Figure 4:**
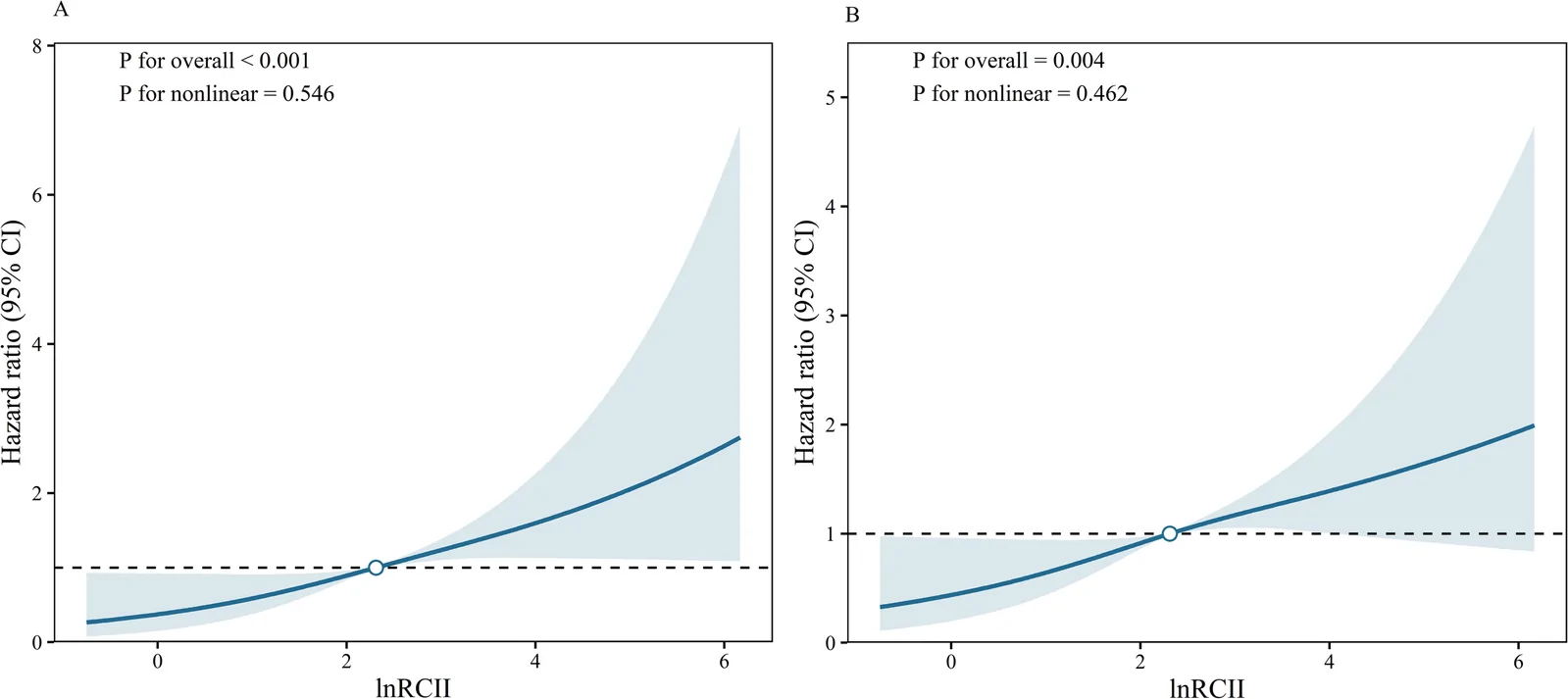
RCS analyses of the associations between lnRCII and recurrent ischemic stroke **(A)** and all-cause mortality **(B)** among patients with AIS treated with intravenous thrombolysis. Models were adjusted for age, gender, baseline NIHSS score, stroke etiology, hypertension, diabetes mellitus, ischemic heart disease, atrial fibrillation, and history of stroke. The reference value was lnRCII = 2.312, corresponding to an HR of 1.

### Sensitivity analysis

In separate sensitivity analyses, after excluding patients with concomitant severe infection or extreme lnRCII values, the association between lnRCII and the risks of recurrent ischemic stroke and all-cause mortality remained statistically significant, with no substantial changes in either effect direction or magnitude (**Supplementary Table S4**).

## Discussion

In this cohort study, we consecutively enrolled patients with AIS who underwent IVT between April 2019 and March 2025, with a median follow-up duration of 36.3 months. The results indicate that elevated lnRCII levels are significantly associated with an increased risk of long-term recurrent ischemic stroke and all-cause mortality. Furthermore, RCS analyses showed no significant evidence of nonlinearity in the associations between lnRCII and either outcome. This finding expands novel evidence supporting the prognostic value of lnRCII in thrombolysed AIS patients and highlights the potential interplay between remnant cholesterol burden, systemic inflammation, and stroke severity in determining long-term clinical outcomes. Previous studies have investigated the associations of RCII with cardiovascular disease and stroke-related outcomes (16–22). Among patients with AIS who underwent endovascular thrombectomy (EVT), E et al. demonstrated that RCII is correlated with poor 90-day functional outcomes (13). More recently, Chen et al. investigated the association between RCII and long-term clinical outcomes in 3, 008 patients with AIS and reported that higher RCII levels were associated with adverse outcomes, including all-cause mortality (23). Unlike this previous study, the present study specifically focused on IVT-treated patients who survived hospitalization and evaluated long-term recurrent ischemic stroke as a separate outcome. Thus, our findings extend existing evidence to a distinct IVT-treated population. Furthermore, evidence from large population-based cohorts further supports the prognostic significance of RCII. In analyses of both the NHANES and CHARLS datasets, higher RCII levels were associated with a substantially increased risk of all-cause mortality, with each standard-deviation increment corresponding to an approximately 25%-30% higher mortality risk after multivariable adjustment (8). These observations indicate that RCII may capture pathophysiological processes linked not only to cerebrovascular disease but also to overall survival.

This study further validated the above findings in patients with AIS who received IVT. The results showed that elevated lnRCII was significantly associated with an increased risk of recurrent ischemic stroke and all-cause mortality, with no significant evidence of nonlinearity. This finding is consistent with previous studies and extends previous evidence regarding the association of RCII with adverse outcomes to patients undergoing IVT. In parallel, findings from a study based on the CHARLS cohort revealed a bidirectional mediation between RC and hs-CRP on stroke risk, with RC accounting for 10.5% of the influence of hs-CRP on stroke risk, and hs-CRP explaining 3.3% of the effect of RC. This suggests a potential bidirectional reinforcing relationship between lipid metabolism disorders and inflammatory activation (12). The findings of this study suggest that elevated RCII, a composite indicator combining remnant cholesterol and inflammatory status, may capture the joint impact of dyslipidemia and systemic inflammation. This may provide biological plausibility for the observed associations with recurrent ischemic stroke and all-cause mortality following IVT.

From a mechanistic perspective, elevated RCII levels are often associated with an increased atherosclerotic burden and heightened inflammatory responses. Specifically, RC not only promotes lipid deposition in the vascular wall (24–27), but also accelerates plaque formation and instability by inducing oxidative stress, endothelial dysfunction, and local inflammatory responses (28–30). CRP (including hs-CRP), on the other hand, reflects the body’s overall inflammatory activation status and may, on this basis, contribute to and exacerbate vascular damage, monocyte chemotaxis, and thrombus-related inflammatory responses (31–34). By integrating RC and CRP, RCII may reflect the combined burden of remnant cholesterol and systemic inflammation. Several biological pathways, including atherosclerotic progression, oxidative stress, endothelial dysfunction, inflammatory activation, impaired microcirculatory perfusion, and ischemia-reperfusion injury, may provide potential explanations for the observed associations between RCII and adverse outcomes after IVT; however, these mechanisms were not directly evaluated in the present study. Previous studies have suggested that the association between RCII and mortality risk may be partially mediated by pathways such as dysglucolipidemia, insulin resistance, and increased blood pressure load. This suggests that RCII does not reflect an abnormality in a single pathway, but rather represents a comprehensive manifestation of the combined effects of multiple cardiovascular and cerebrovascular risk factors (35, 36). However, the prognosis following IVT is influenced by various factors, including stroke severity, etiological classification, and inflammatory levels during the peri-treatment period; a single indicator is often insufficient to fully reflect the overall risk (37, 38). In the component analyses, lnCRP remained significantly associated with all-cause mortality after adjustment for RC, whereas RC was not significantly associated with mortality, suggesting that the association between RCII and mortality may be largely driven by its inflammatory component. However, these analyses do not establish synergistic effects or demonstrate incremental predictive value of RCII over its individual components. The findings of this study further support the important role of inflammation and dyslipidemia in the long-term prognosis of AIS.

In exploratory subgroup analyses, potential heterogeneity by age and hypertension was observed in the association of lnRCII with all-cause mortality, whereas no statistically significant interactions were observed for recurrent ischemic stroke. However, these findings should be interpreted cautiously because multiple subgroup comparisons were performed without adjustment for multiplicity. This pattern may partly reflect differences in baseline risk profiles, comorbidity burden, and competing risks between younger and older patients, which could attenuate the relative prognostic contribution of lnRCII in the elderly population.

Overall, this study has certain clinical significance. First, the RCII is calculated using routine laboratory test parameters, making it easy to obtain and relatively inexpensive, and thus highly accessible in clinical practice. Second, RCII simultaneously reflects remnant cholesterol burden and inflammatory status and may serve as a composite marker associated with long-term outcomes after IVT. For patients with AIS undergoing IVT, early identification of those with elevated RCII levels may help identify individuals with an increased risk of long-term recurrent ischemic stroke and mortality, although whether RCII-guided risk assessment can improve secondary prevention and long-term outcomes requires further prospective investigation.

While this study has yielded valuable findings, it also has several limitations. First, the single-center observational design and restriction to IVT-treated patients who survived the index hospitalization may limit the generalizability of our findings. The exclusion of patients with missing data or unavailable follow-up information may have introduced selection bias, while the exclusion of in-hospital deaths and patients receiving bridging therapy may further limit generalizability. Therefore, our findings should be interpreted as associations among IVT-treated patients who survived hospitalization rather than the entire IVT-treated population. Despite multivariable adjustment and sensitivity analyses, residual confounding cannot be excluded. Second, the RCII was based exclusively on initial test results at baseline. No repeated measurements of lipid or inflammatory levels were performed during follow-up. As a result, the potential influence of dynamic changes in RCII on patient prognosis could not be examined. Furthermore, post-thrombolysis CRP levels cannot distinguish pre-existing chronic inflammation from the acute inflammatory response to the index stroke or concurrent infection, and reverse causation cannot be excluded. Although all 20 clinically documented severe infections occurred after baseline blood sampling, subclinical infection or a preceding inflammatory response at the time of sampling cannot be excluded. Furthermore, excluding patients based on subsequent clinical complications in the sensitivity analysis may have introduced selection bias. In addition, longitudinal data on secondary prevention therapies, smoking cessation, and blood pressure control were not systematically collected and therefore could not be included in the analyses. Third, some patients with recurrent ischemic stroke may not seek medical attention due to mild or asymptomatic symptoms, which may lead to an underestimation of the observed recurrent ischemic stroke risk. Moreover, most patients did not undergo repeat vascular imaging after IVT; therefore, standardized post-thrombolysis recanalization status was unavailable and could not be accounted for in the analyses. Finally, because the study included only patients treated with IVT and lacked a non-IVT comparison group, the observed associations cannot be specifically attributed to IVT itself.

## Conclusion

In summary, among patients with AIS who survived hospitalization following IVT, elevated RCII was independently associated with an increased risk of long-term recurrent ischemic stroke and all-cause mortality. RCII may serve as a composite marker associated with long-term outcomes after IVT. Nevertheless, further validation in large-scale, multicenter prospective studies is warranted before its routine clinical application.

## Data Availability

The raw data supporting the conclusions of this article will be made available by the authors, without undue reservation.
