# Supplementary material for: Association of the remnant cholesterol inflammation index with long-term outcomes among patients with acute ischemic stroke treated with intravenous thrombolysis
Source: Front Endocrinol (Lausanne). 2026 Nov 5;17:1984138. doi: 10.3389/fendo.2026.1984138 (PMC13755835; doi:10.3389/fendo.2026.1984138)
Supplement: Supplementary file 1 [file Table1.docx]

**Table S1 Fine–Gray Competing-Risk Regression Analysis of the Association Between lnRCII and Recurrent Ischemic Stroke**

| **Variables** | **Crude sHR (95% CI)** | **P value** | **Adjusted sHR (95% CI)** | **P value** |
| --- | --- | --- | --- | --- |
| lnRCII | 1.436(1.249-1.651) | < 0.001 | 1.341(1.161-1.550) | < 0.001 |
| lnRCII, Quartiles |  |  |  |  |
| Q1 | Ref |  | Ref |  |
| Q2 | 2.079(1.005-4.300) | 0.048 | 2.000 (0.971-4.122) | 0.060 |
| Q3 | 2.967(1.495-5.888) | 0.002 | 2.577 (1.280-5.185) | 0.008 |
| Q4 | 3.761(1.937-7.302) | < 0.001 | 3.094 (1.596-6.000) | < 0.001 |

The competing-risk analysis included 748 patients, with 101 recurrent ischemic stroke events and 88 deaths before recurrent ischemic stroke treated as competing events. Adjusted models included age, gender, baseline NIHSS score, stroke etiology, hypertension, diabetes mellitus, ischemic heart disease, atrial fibrillation, and history of stroke. sHR, subdistribution hazard ratio; CI, confidence interval; RCII, remnant cholesterol inflammation index.

**Table S2** **Associations of lnRCII and Its Components in Relation to Recurrent Ischemic Stroke**

| **Model** | **Variable** | **HR (95% CI)** | **P value** |
| --- | --- | --- | --- |
| lnRCII | lnRCII | 1.373 (1.174-1.605) | < 0.001 |
| RC | RC | 1.016 (1.007-1.024) | < 0.001 |
| lnCRP | lnCRP | 1.290 (1.098-1.516) | 0.002 |
| RC+lnCRP | RC | 1.018 (1.009-1.027) | < 0.001 |
|  | lnCRP | 1.340 (1.136-1.580) | < 0.001 |

All models were adjusted for age, gender, baseline NIHSS score, stroke etiology, hypertension, diabetes mellitus, ischemic heart disease, atrial fibrillation, and history of stroke. In the RC + lnCRP model, RC and lnCRP were entered simultaneously.

Continuous effect estimates are expressed per one-unit increase in lnRCII, per 1 mg/dL increase in RC, and per one-unit increase in lnCRP, respectively.

CI, confidence interval; CRP, C-reactive protein; HR, hazard ratio; NIHSS, National Institutes of Health Stroke Scale; RC, remnant cholesterol; RCII, remnant cholesterol inflammation index.

**Table S3 Associations of lnRCII and Its Components in Relation to All-Cause Mortality**

| **Model** | **Variable** | **HR (95% CI)** | **P value** |
| --- | --- | --- | --- |
| lnRCII | lnRCII | 1.275 (1.107-1.469) | < 0.001 |
| RC | RC | 1.004 (0.997-1.012) | 0.282 |
| lnCRP | lnCRP | 1.301 (1.128-1.501) | < 0.001 |
| RC+lnCRP | RC | 1.006 (0.998-1.014) | 0.164 |
|  | lnCRP | 1.315 (1.138-1.520) | < 0.001 |

All models were adjusted for age, gender, baseline NIHSS score, stroke etiology, hypertension, diabetes mellitus, ischemic heart disease, atrial fibrillation, and history of stroke. In the RC + lnCRP model, RC and lnCRP were entered simultaneously.

Continuous effect estimates are expressed per one-unit increase in lnRCII, per 1 mg/dL increase in RC, and per one-unit increase in lnCRP, respectively.

CI, confidence interval; CRP, C-reactive protein; HR, hazard ratio; NIHSS, National Institutes of Health Stroke Scale; RC, remnant cholesterol; RCII, remnant cholesterol inflammation index.

**Supplementary Table S4 Sensitivity Analyses of the Associations of lnRCII With Recurrent Ischemic Stroke and All-Cause Mortality**

| **Variables** | **Outcome** | **N** | **Events** | **HR (95% CI)** | **P value** |
| --- | --- | --- | --- | --- | --- |
| Exclude severe infection | Recurrent ischemic stroke | 728 | 97 | 1.465 (1.220-1.758) | < 0.001 |
|  | All-cause mortality | 728 | 112 | 1.249 (1.059-1.473) | 0.008 |
| Exclude extreme lnRCII | Recurrent ischemic stroke | 744 | 101 | 1.436 (1.217-1.695) | < 0.001 |
|  | All-cause mortality | 744 | 117 | 1.254 (1.080-1.457) | 0.003 |

CI, confidence interval; HR, hazard ratio; RCII, remnant cholesterol inflammation index. Models were adjusted for age, gender, baseline NIHSS score, stroke etiology, hypertension, diabetes mellitus, ischemic heart disease, atrial fibrillation, and history of stroke. Extreme lnRCII values were defined using the 1.5 × interquartile range rule (lnRCII >5.788).
